# Klotho protein contributes to cardioprotection during ischaemia/reperfusion injury

**DOI:** 10.1111/jcmm.15293

**Published:** 2020-04-21

**Authors:** Agnieszka Olejnik, Anna Krzywonos‐Zawadzka, Marta Banaszkiewicz, Iwona Bil‐Lula

**Affiliations:** ^1^ Division of Clinical Chemistry and Laboratory Hematology Department of Medical Laboratory Diagnostics Faculty of Pharmacy with Division of Laboratory Diagnostics Wroclaw Medical University Wroclaw Poland

**Keywords:** cardiac markers, cardioprotection, ischaemia/reperfusion injury, Klotho protein

## Abstract

Restoration of blood flow to ischaemic heart inflicts ischaemia/reperfusion (I/R) injury, which manifests in metabolic and morphological disorders. Klotho is a protein with antioxidative and antiapoptotic activity, and is involved in the regulation of inflammation and fibrosis. The aim of the current research was to determine the role of Klotho in the heart subjected to I/R injury, as well as to study Klotho as a potential cardioprotective agent. Human cardiomyocytes and Wistar rat hearts perfused using Langendorff method subjected to I/R have been used. Hemodynamic parameters of heart function, markers of I/R injury, and gene and protein expression of Klotho were measured. Human cardiomyocytes were also incubated in the presence of recombinant Klotho protein, and the viability of cells was measured. There was a higher expression of Klotho gene and protein synthesis in the cardiomyocytes subjected to I/R injury. The compensatory production and release of Klotho protein from cardiac tissue during I/R were also shown. The treatment of cardiomyocytes subjected to I/R with Klotho protein resulted in increased viability and metabolic activity of cells. Thus, Klotho contributes to compensatory mechanism during I/R, and could be used as a marker of injury and as a potential cardiopreventive/cardioprotective agent.

## 1 INTRODUCTION

The standard procedure for treatment of myocardial infarction (MI) is coronary reperfusion.^1^, ^2^ Whereas reperfusion is mandatory in the therapeutic approach to ischaemia, revascularization and restoration of the blood flow to ischaemic myocardium inflicts additional damage—ischaemia/reperfusion (I/R) injury. In turn, I/R injury causes metabolic, morphological and contractile disorders, leading to irreversible microvascular damage or myocardial stunning.^3^, ^4^ An excessive formation of reactive oxygen species (ROS), degradation of contractile proteins by proteolytic enzymes and necrotic cell death are important factors contributing to the pathogenesis of I/R injury.^3^, ^5^

Klotho (KL) is a membrane‐bound or soluble antiaging protein with antioxidative and antiapoptotic activity. The expression of Klotho gene is observed mainly in the kidneys and brain.^6^, ^7^ It was proved that soluble KL is involved in the regulation of oxidative stress, inflammation and fibrosis.^8^, ^9^, ^10^, ^11^ Experimental studies of exogenous Klotho protein administration have apparently affirmed its protective role in several cell and animal models.^10^, ^12^, ^13^, ^14^ In favour of a rationale that supports the protective role of Klotho, we decided to examine Klotho as a potential preventive/therapeutic agent in I/R heart injury.

The aim of this study was to investigate the role of Klotho protein in the heart tissue subjected to I/R injury, as well as to study Klotho as a potential cardioprotective agent.

## 2 MATERIALS AND METHODS

In this study, animal tissues obtained from our other project were used. All animal procedures conformed to the Guide to the Care and Use of Experimental Animals published by the Polish Ministry of Science and Higher Education. This investigation was approved by the Ethics Committee for Experiments on Animals at the Ludwik Hirszfeld Institute of Immunology and Experimental Therapy Polish Academy of Sciences, Wroclaw, Poland (Resolution 14/2016 of 20 April 2016).

### 2.1 Cell culture

The primary human cardiac myocytes (HCM) were purchased from ScienCell Research Laboratories. The cells were cultured at 37°C in a water‐saturated, 5% CO_2_ atmosphere in Dulbecco's Modified Eagle's Medium (Sigma‐Aldrich) containing Cardiac Myocyte Growth Supplement (ScienCell Research Laboratories), 5% foetal bovine serum, 100 U/mL penicillin and 100 μg/mL streptomycin (all from Sigma‐Aldrich). Cells were passaged at 90% confluence using 0.25% trypsin‐EDTA (Sigma‐Aldrich).

### 2.2 The protocol of in vitro chemical I/R injury of cardiomyocytes

**FIGURE 1:**
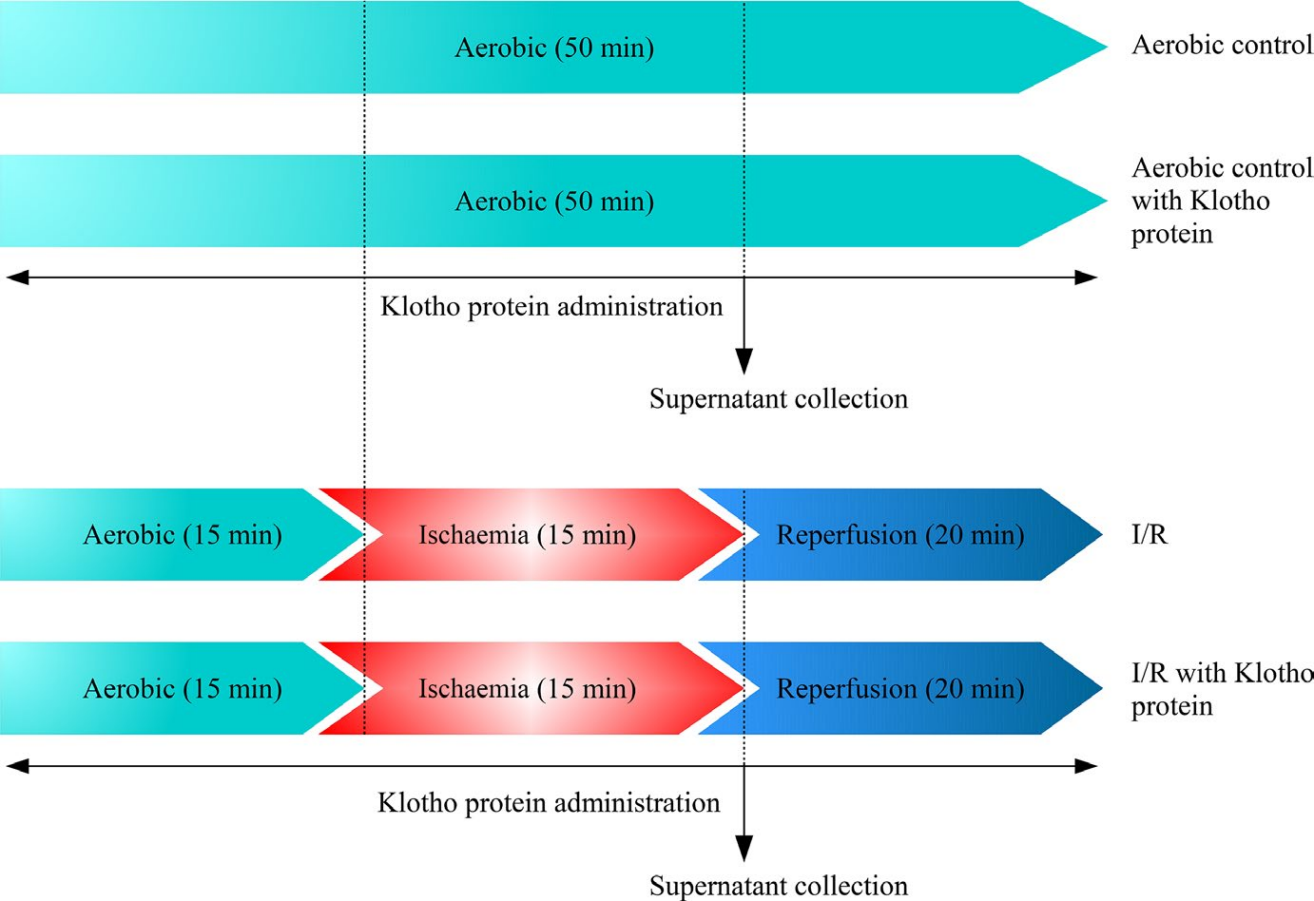
Experimental protocol for in vitro chemical IR injury of cardiomyocytes with and without Klotho administration. I/R, ischaemia/reperfusion

Cardiac myocytes in culture underwent in vitro chemical I/R in accordance with the guidelines for experimental models of myocardial ischaemia and infarction.^15^ The scheme of the experimental protocol is shown in Figure 1. Briefly, HCM underwent 15 minutes of aerobic stabilization, 15 minutes of in vitro chemical ischaemia and 20 minutes of reperfusion,^16^ in the presence and absence of 1 µg/mL ^17^ of Recombinant Human Klotho Protein (R&D Systems, 5334‐KL‐025). The aerobic stabilization and reperfusion was performed in 4‐(2‐hydroxyethyl)‐1‐piperazineethanesulfonic acid (HEPES) buffer (5.5 mmol/L HEPES, 63.7 mmol/L CaCl_2_, 5 mmol/L KCl, 2.1 mmol/L MgCl_2_, 5.5 mmol/L glucose and 10 mmol/L taurine) containing additional 55 μmol/L CaCl_2_ and 0.75 mg/mL BSA. In an in vitro chemical ischaemia group, cells were incubated in HEPES buffer containing 4.4 mmol/L 2‐deoxyglucose (to inhibit glycolysis) and 4.0 mmol/L sodium cyanide (an inhibitor of cellular respiration).^16^ The optimal duration of ischaemia (15 minutes) was established experimentally by measurement the activity of lactate dehydrogenase (LDH) released from cells as a marker of cell injury (data not shown). In the I/R group, after 15 minutes of aerobic stabilization in HEPES buffer at RT, the buffer was removed by centrifugation (1 minute 1500 g) and the cell pellet was resuspended in ischaemia buffer and incubated for 15 minutes at RT. Then, the cells were centrifuged for 1 minutes at 1500 *g*, the buffer was removed and cells were incubated in aerobic conditions with HEPES buffer for 20 minutes at RT (reperfusion). After reperfusion, the buffer was removed by centrifugation at 1500 *g* for 5 minutes, and the cell pellet was homogenized. The cells from aerobic control group were incubated aerobically for 50 minutes in HEPES buffer at RT. In the Klotho experimental groups (aerobic + Klotho, I/R + Klotho), the cells underwent experimental protocol in the presence of Klotho protein (1 µg/mL final concentration) during whole procedure. Myocytes from aerobic group subjected to Klotho protein were tested to check the cytotoxicity of Klotho.

### 2.3 Cell homogenization

Cells underwent three cycles of freezing (in liquid nitrogen) and thawing (at 37°C) in the homogenization buffer (50 mmol/L Tris‐HCl [pH 7.4] containing 3.1 mmol/L sucrose, 1 mmol/L DTT, 10 µg/mL leupeptin, 10 µg/mL soybean trypsin inhibitor, 2 µg/mL aprotinin and 0.1% Triton X‐100). Then, myocytes were homogenized mechanically on ice (three times for 10 seconds) with a hand‐held homogenizer. Supernatants were obtained by centrifugation at 10 000 *g* for 5 minutes at 4°C and then were transferred into a fresh tube. The homogenates were stored at −80°C for further experiments.

### 2.4 Klotho mRNA expression

Total RNA from HCM was isolated with TRIZOL reagent (Thermo Fisher Scientific) according to the manufacturer's instruction. Microultraviolet (UV) spectrophotometer (NanoDrop Lite, Thermo Scientific) was used to evaluate the concentration and purity of RNA. Reverse transcription of pure RNA samples (200 ng) was conducted in order to prepare cDNA with iScript cDNA Synthesis Kit (Bio‐Rad) according to the instructions provided. Briefly, the reverse transcription was carried out at 42°C for 30 minutes and inactivated at 85°C for 5 minutes. The expression level of glucose‐6‐phosphate dehydrogenase (G6PD) was used as internal reference for Klotho. To analyse expression of Klotho gene in a ratio to G6PD gene, real‐time quantitative PCR (RT‐qPCR) and CFX96 Real‐Time System (Bio‐Rad) were used. Briefly, the final volume of the reaction mix was 30 μL and included iTag Universal Sybr Green Supermix with ROX (Bio‐Rad), forward and reverse primers (250 nmol/L final conc.), water and cDNA (40 ng). The primers for Klotho were designed by us and synthesized by TIB Molbiol (TIB Molbiol, Berlin, Germany). The sequences of primers 5′–3′ are as follows: Human Klotho F: AGGGTCCTAGGCTGGAATGT and Human Klotho R: CCTCAGGGACACAGGGTTTA. The amount of particular mRNAs relative to G6PD was calculated as 2^−ΔCt^. 2^−ΔCt^ was equal to the relative transcriptional mRNA level of Klotho gene in cells that were exposed to aerobic conditions and cells subjected to I/R.

### 2.5 Klotho protein concentration in cardiomyocytes

Klotho protein concentration in cell homogenates was measured quantitatively using Sandwich Human Klotho ELISA Kit from Biorbyt (Biorbyt Ltd.), according to manufacturer's instruction. After resetting the blank well, the optical density (OD) (at 450 nm) of each well was measured by Spark multimode microplate reader (Tecan Trading AG). Klotho protein concentration was normalized to total protein concentration in cell homogenates and expressed in pg/μg protein. The concentration of Klotho was compared in cells that were exposed to aerobic conditions and cells subjected to I/R.

### 2.6 Immunofluorescence staining of cardiomyocytes

The cells were cultured in 24‐well cell culture plate (Greiner Bio‐One GmbH) at a density of 1 × 10^5^ cells/well. When the cell confluence reached approximately 90%, myocytes underwent in vitro chemical I/R injury in the cell culture plate (please see the protocol shown in Figure 1). After removing the cell culture medium and washing with phosphate buffered saline (PBS), cells were subjected to fixation according to the following steps: 500 μL/well of 4% paraformaldehyde was added and left to fix at RT for 15 minutes. After three time of PBS rinse, myocytes were incubated with blocking buffer (1% BSA, 10% goat serum, 3 M glycine in 0.1% Tween‐PBS) for 1 hour at RT. Primary antibody rabbit anti‐Klotho 1:1000 (Sigma‐Aldrich, SAB3500604) was incubated at 4°C overnight and then washed with PBS. The secondary antibody donkey anti‐rabbit IgG 1:500 (abcam, ab98488) labelled with DyLight^®^ 488 was added secondly and incubated at RT for 45 minutes. To visualize cells’ nuclei, myocytes were stained with DAPI 1:1000 (4',6‐diamidino‐2‐phenylindole, Sigma‐Aldrich) for 15 minutes in the dark and rinsed with PBS. ZOE Fluorescent Cell Imager (Bio‐Rad) was used to estimate the bright green fluorescence for Klotho and blue fluorescence for DAPI. Image J 1.52a software (NIH) was used to analyse the area of fluorescence of each image. The number of cells was assessed by measuring the fluorescence of cells’ nuclei stained by DAPI (blue fluorescence). The cell surface expression of Klotho was assessed by measuring the green fluorescence of fixed cells stained with anti‐Klotho antibodies and normalized to the number of cells (blue fluorescence). Data were collected from three independent images trials for each experiment and shown as AU. The cell surface expression of Klotho was compared in cells that were exposed to aerobic conditions and cells subjected to I/R.

### 2.7 Cytotoxicity assay for cardiomyocytes

To assess an influence of Klotho protein on cell viability, the CytoTox‐Glo™ Cytotoxicity Assay (Promega) was performed.^18^ The assay uses a luminogenic peptide substrate to measure ‘dead‐cell protease activity’, which is released from cells that have lost membrane integrity. The assay selectively detects dead cells, because substrate cannot cross the intact membrane of live cells and does not generate signal from the live‐cell population. In brief, cells were seeded in 96‐well plate at a density of 1 × 10^4^ cells/well for 24 hours and then subjected to in vitro chemical I/R injury in the presence and absence of 1 µg/mL Klotho protein (please see the protocol shown in Figure 1). The luminescence was recorded on Spark multimode microplate reader (Tecan Trading AG). The data were normalized to cell confluence and expressed in relative light units (RLU). The viability of myocytes was based on the number of death cells in each well and compared to cells that were exposed to aerobic conditions and cells subjected to I/R and I/R with the addition of recombinant Klotho.

### 2.8 The metabolic activity of cardiomyocytes

The metabolic activity of cells subjected to I/R was assessed using the vital inclusion dye fluorescein diacetate (FDA) and the vital exclusion dye DAPI.^19^ In not injured cells, appropriate esterases are present in an active form. Viable cells have the capability to incorporate the non‐polar, non‐fluorescent FDA and rapidly hydrolyse it into fluorescein using acetyl esterase. Fluorescein is a polar, fluorescent compound which is retained within the cell. In the damaged cells, esterified fluorescein is rapidly diffuse from cell to liquid medium and cell visualization is not allowed. Non‐viable cells are susceptible to DNA staining with DAPI, where fluorescence is produced by interacting with the nucleus. On the basis of these principals, FDA can be easily used in a double staining procedure in combination with DAPI.^20^, ^21^ Briefly, myocytes were seeded in 96‐well plate at a density of 2 × 10^4^ cells/well for 24 hours and then subjected to in vitro chemical I/R injury in the presence and absence of 1 µg/mL Klotho protein (please see the protocol shown in Figure 1). The cells were washed three times with PBS and stained with 5 μg/mL FDA (Sigma‐Aldrich) and 1:1000 DAPI (Sigma‐Aldrich) for 15 minutes in the dark. ZOE Fluorescent Cell Imager (Bio‐Rad) was used to estimate the bright green fluorescence for FDA and blue fluorescence for DAPI. Cells were considered metabolically active when stained with FDA and excluded DAPI, whereas cells that excluded FDA and stained with DAPI were considered necrotic.^19^ Image J 1.52a software (NIH) was used to analyse the area of fluorescence of each image. To determine the metabolic activity of cells, green fluorescence (live cells) was normalized to the total number of cells (green + blue fluorescence) in each experiment.^19^ Data were collected from three independent images trials for each experiment and shown as AU. The metabolic activity was compared in cells that were exposed to aerobic conditions and cells subjected to I/R and I/R with the addition of recombinant Klotho.

### 2.9 Isolated rat hearts perfused with the Langendorff method

To determine the expression of Klotho protein at the tissue level, isolated rat heart model was used. Ten‐ to 11‐week‐old male Wistar rats (250‐350 g) were treated with buprenorfin (0.05 mg/kg, ip) and anaesthetized with sodium pentobarbital (0.5 mL/kg i.p.). The hearts were rapidly excised from animals and rinsed by immersion in ice‐cold Krebs‐Henseleit Buffer containing 118 mmol/L NaCl, 4.7 mmol/L KCl, 1.2 mmol/L KH_2_PO_4_, 1.2 mmol/L MgSO_4_, 3.0 mmol/L CaCl_2_, 25 mmol/L NaHCO_3_, 11 mmol/L glucose and 0.5 mmol/L EDTA, pH 7.4. Spontaneously beating isolated hearts were cannulated by the aorta on a Langendorf system (EMKA Technologies) and perfused at a constant pressure of 60 mm Hg with Krebs‐Henseleit Buffer at pH 7.4, at 37°C and gassed continuously with 5% CO_2_/95% O_2_. After stabilization, the hearts were subjected to protocol of global I/R injury. As haemodynamic end‐points of cardioprotection,^22^ coronary flow (CF), heart rate (HR), left ventricular developed pressure (LVDP), left ventricular end‐diastolic pressure (PED) and intraventricular pressure (dp/dt) were determined using an EMKA recording system with IOX2 software (EMKA Technologies). At the end of protocol, isolated hearts were immediately immersed in liquid nitrogen and stored at −80°C before further investigations.

### 2.10 Global ischaemia/reperfusion of isolated rat hearts

Hearts in I/R group were subjected to aerobic perfusion for 25 minutes. After oxygenation, the heart perfusion was stopped (by cessation of the buffer flow) and hearts underwent global no‐flow ischaemia for 22 minutes.^16^ Then, hearts were perfused in aerobic conditions for 30 minutes (reperfusion). The hearts from aerobic control group were perfused aerobically for 77 minutes. At the beginning of reperfusion (47 minutes), 15 mL of coronary effluents was collected for biochemical studies. To determine cardiac mechanical function, the recovery of rate pressure product (RPP) was expressed as the product of HR and LVPD and evaluated at 25 minutes of experiment (the end of aerobic perfusion) and at 77 minutes (the end of reperfusion). Then, the analysis of functional and biochemical changes between aerobic and I/R group was performed.

### 2.11 Preparation of heart homogenates

Hearts previously frozen at −80°C were crushed using a mortar and pestle in liquid nitrogen and then homogenized in ice‐cold homogenization buffer containing: 50 mmol/L Tris‐HCl (pH 7.4), 3.1 mmol/L sucrose, 1 mmol/L dithiothreitol, 10 mg/mL, leupeptin, 10 mg/mL soybean trypsin inhibitor, 2 mg/mL aprotinin and 0.1% Triton X‐100. The homogenate was centrifuged at 10 000*g* at 4°C for 15 minutes, and the supernatant was collected and stored at − 80°C for further experiments.

### 2.12 Qualitative and quantitative analysis of Klotho protein in the heart tissue and coronary effluents

The expression of Klotho protein in the rat heart tissue was determined qualitatively by immunoblot and quantitatively by ELISA test in aerobic control and I/R groups. The content of Klotho protein in coronary effluents was determined by immunoblot. In the Western blot analysis, an aliquot of 20 μg of total proteins from heart homogenates and an aliquot of 40 μL of coronary effluents were separated on 10% SDS‐PAGE and then transferred onto nitrocellulose membrane (Bio‐Rad), which were then blocked with 5% BSA. Klotho protein was detected with rabbit anti‐Klotho polyclonal antibody 1:1000 (Sigma‐Aldrich, SAB3500604) and secondary goat anti‐rabbit IgG horseradish peroxidase conjugate antibody 1:5000 (Thermo Fisher Scientific, #31460). The membranes were developed with the ClarityTM Western ECL substrate (Bio‐Rad) using the ChemiDocTM MP System. Target protein bands in coronary effluents were quantified using Quantity One Software (Bio‐Rad). 120‐130 kDa Recombinant Human Klotho (R&D Systems, 5334‐KL‐025) and 65‐70 kDa Recombinant Human Klotho (Sigma‐Aldrich, SRP3102) were used as positive controls. The content of Klotho protein in coronary effluents was expressed as AU and normalized to CF. For quantitative evaluation of Klotho protein in the heart tissue, Rat Klotho ELISA Kit (Cusabio Technology LLC) was used according to manufacturer's instruction. Each sample was assayed in duplicate. Klotho protein concentration was normalized to total protein concentration in tissue homogenates and expressed in pg/μg protein. The level of Klotho was compared in hearts that were exposed to aerobic conditions and hearts subjected to I/R.

### 2.13 Assessment of LDH level

To determine the activity of LDH in coronary effluents, Lactate Dehydrogenase Activity Assay Kit (Sigma‐Aldrich) according to manufacturer's instruction was used. LDH is a stable cytosolic enzyme that is released upon membrane damage/permeability or cell lysis and serves as a marker of cell damage. LDH level was normalized to CF and compared in coronary effluents of hearts that were exposed to aerobic conditions and hearts subjected to I/R.

### 2.14 Assessment of the number of death cells in rat hearts

To assess an influence of I/R injury on isolated rat hearts, the number of death cells using the CytoTox‐Glo™ Cytotoxicity Assay (Promega) according to manufacturer's instruction was tested. The measurement of extracellular activity of dead‐cell protease was performed in coronary effluents and normalized to CF. The number of death cells was compared in hearts that were exposed to aerobic conditions and hearts subjected to I/R.

### 2.15 Determination of protein concentration

Bradford method was used to determine protein concentration in the cardiac tissue and cell homogenates. BSA (heat shock fraction, ≥98%, Sigma‐Aldrich) served as the protein standard. For measuring total protein concentration, Bio‐Rad Protein Assay Dye Reagent (Bio‐Rad) and Spark multimode microplate reader (Tecan Trading AG) were used.

### 2.16 Statistical analysis

Experimental data were analysed using GraphPad Prism 6 software (GraphPad Software). Shapiro‐Wilk normality test or Kolmogorov‐Smirnov test was used to assess normality of variances changes. The Student's *t* test or Mann‐Whitney *U* test was used for comparison between two groups of measurement data. ANOVA or non‐parametric test with post hoc tests for multiple groups was used. Correlations were assessed using Pearson's or Spearman's test, as appropriate. Results were expressed as mean ± SEM, and a value of *P* < .05 was regarded as statistically significant.

## 3 RESULTS

### 3.1 Enhanced expression of Klotho in human cardiomyocytes subjected to I/R injury

**FIGURE 2:**
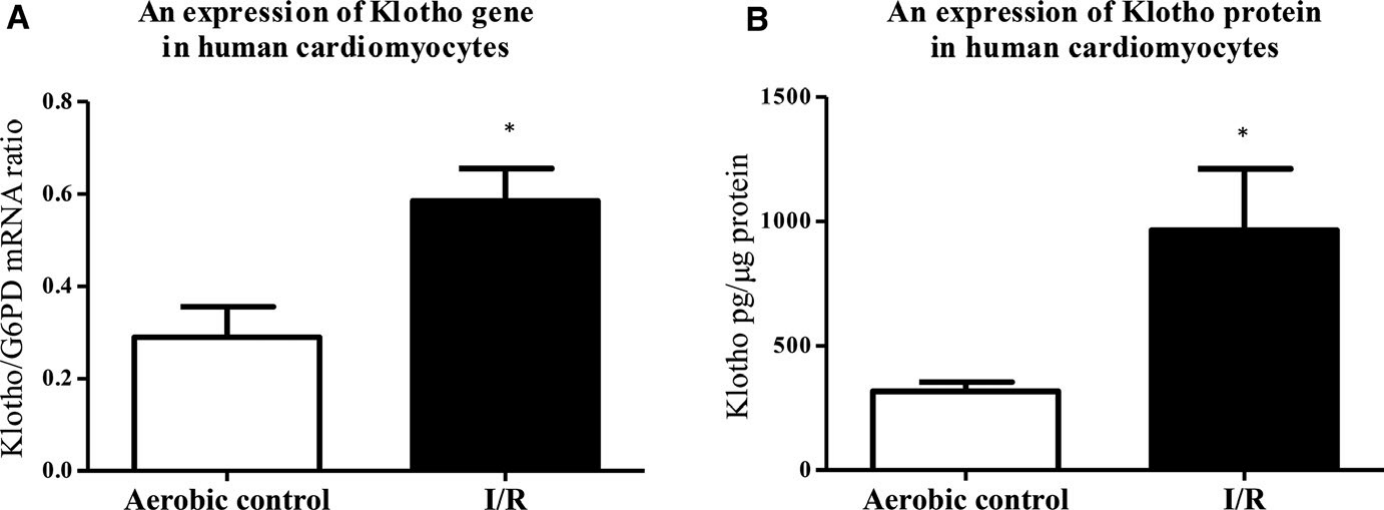
Klotho in human cardiomyocytes subjected to I/R injury. A, An expression of Klotho gene in human cardiomyocytes was examined by RT‐qPCR and normalized to G6PD, n = 6‐12. B, Quantitative analysis of Klotho protein in human cardiomyocytes by ELISA. Klotho protein concentration was normalized to total protein concentration, n = 18. G6PD, glucose‐6‐phosphate dehydrogenase; I/R, ischaemia/reperfusion; *P < .05 vs aerobic control; all data are expressed as mean ± SEM

**FIGURE 3:**
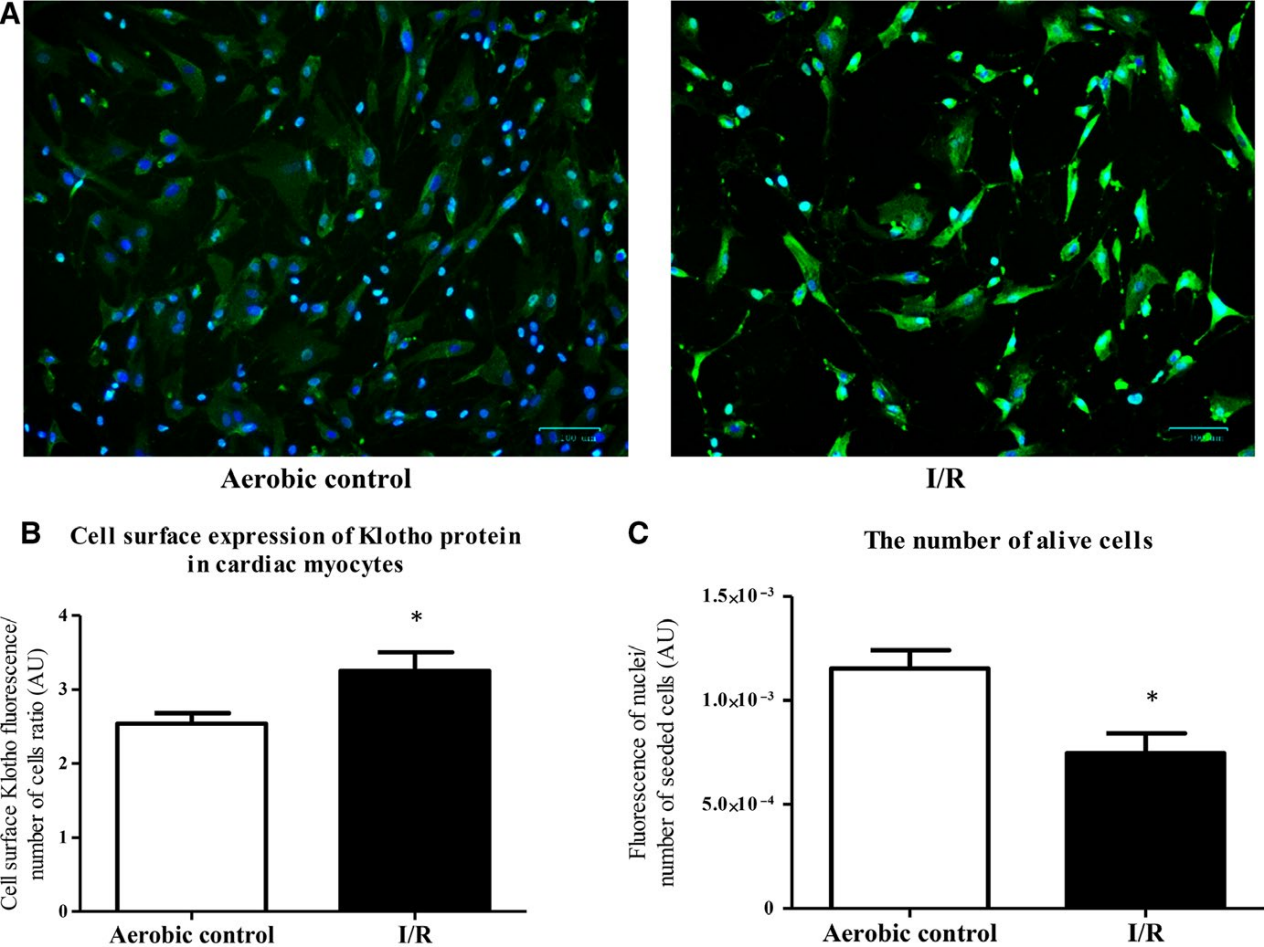
Cell surface expression of Klotho protein in human cardiomyocytes. A, Immunofluorescence staining of human cardiomyocytes for Klotho (green fluorescence) and DAPI for nuclei (blue fluorescence). B, The cell surface expression of Klotho in cardiomyocytes was assessed by measuring the green fluorescence of fixed cells stained with anti‐Klotho antibodies and DyLight 488 and normalized to the total number of cells (blue fluorescence). C, The number of cells was assessed by measuring the fluorescence of cells' nuclei stained by DAPI (blue fluorescence). Graph bars show the average of total fluorescence of cells in each experiment. Magnification 200×; scale bar = 100 μm; AU, arbitrary unit; DAPI, 4',6‐diamidino‐2‐phenylindole; I/R, ischaemia/reperfusion; *P < .05 vs aerobic control; n = 6; all data are expressed as mean ± SEM

The analysis showed significantly higher expression of Klotho mRNA in myocytes subjected to I/R in comparison with aerobic control group (Figure 2A). Then, we checked if increased expression of Klotho mRNA was accompanied by increased protein synthesis. As shown in Figure 2B, the level of Klotho protein was significantly higher in cells that underwent I/R. We have also observed significantly higher cell surface expression of Klotho protein in the cardiomyocytes subjected to I/R, tested by the immunofluorescence staining (Figure 3A,B). The number of cells in I/R group was significantly lower in comparison with aerobic control, due to damage and apoptosis (Figure 3C).

### 3.2 Decreased mechanical function due to heart injury during I/R

**FIGURE 4:**
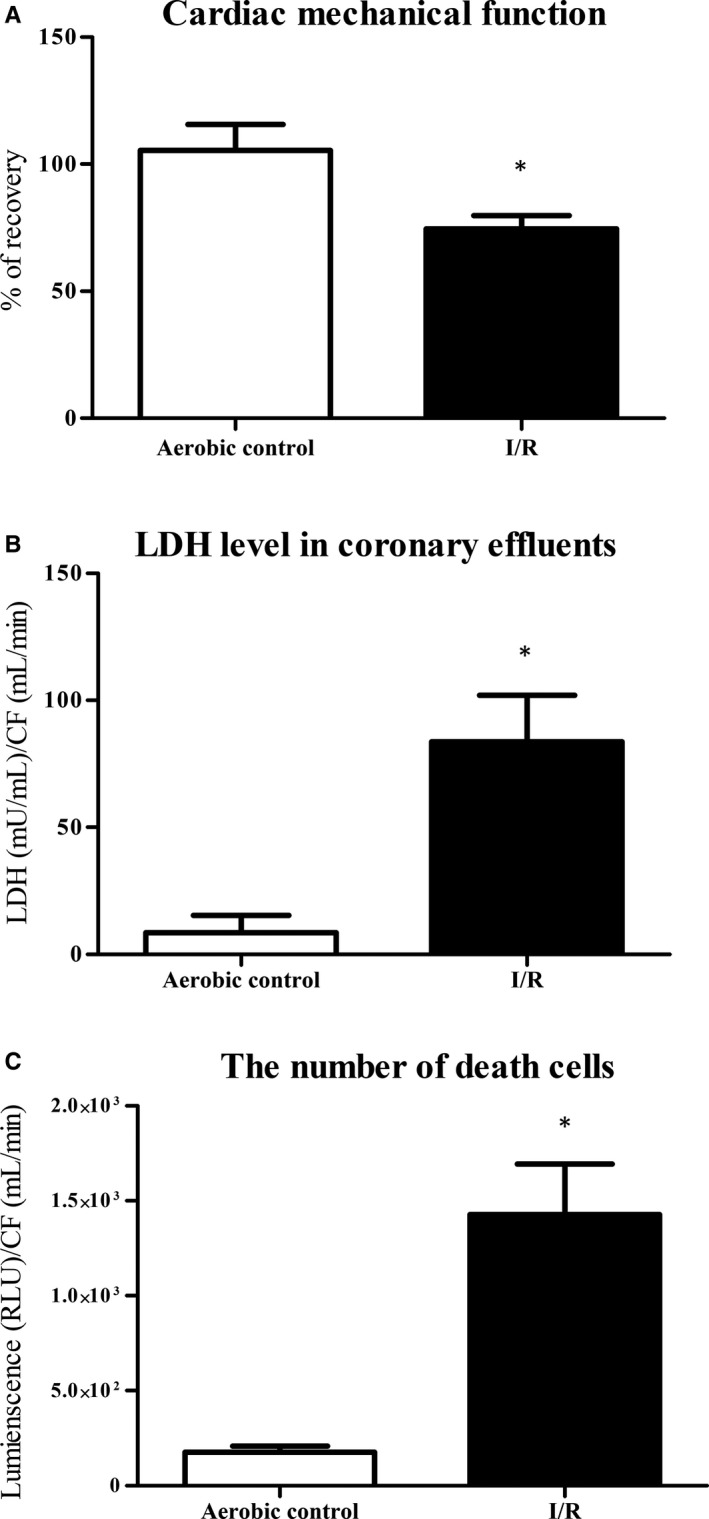
An effect of global I/R injury on isolated rat hearts. A, Recovery of mechanical function of the hearts. Cardiac mechanical function was expressed as the product of heart rate and left ventricular developed pressure—rate pressure product (RPP). RPP was measured at the end of aerobic perfusion (25 min) and at the end of reperfusion (77 min). B, LDH level in coronary effluents as a marker of cell death. LDH level was normalized to CF. C, The number of death cells in rat hearts by luminescent cytotoxicity assay. The data were normalized to CF and expressed in RLU. CF, coronary flow; LDH, lactate dehydrogenase; mU/mL, milli international enzyme units per millilitre; RLU, relative light units; *P < .05 vs aerobic control; mean ± SEM; n = 4‐8

Cardiac mechanical function was decreased in hearts subjected to I/R in comparison with aerobically perfused hearts, showing increased heart injury (Table 1; Figure 4A). The release of LDH from the hearts (marker of cell injury) was significantly higher in I/R group, which showed damage of cardiac cells due to I/R (Figure 4B). The activity of death cell proteases tested by cytotoxicity assay was significantly higher in coronary effluents from hearts subjected to I/R in comparison with aerobic group, indicating increased number of death cells in hearts injured by I/R (Figure 4C).

**TABLE 1:** Cardiac mechanical function of isolated rat hearts (mean ± SEM)

Parameter	Aerobic control	I/R	*P*
HR (bpm)^a^	305.3 ± 6.5	70.7 ± 36.0*	.0002
CF (mL/min)^b^	12.6 ± 1.4	3.2 ± 1.1*	.0024
dP/dt_max_ (mm Hg/s)^a^	1543 ± 117	1300 ± 51	.0657
dP/dt_min_ (mm Hg/s)^a^	−1451 ± 113	−1126 ± 78*	.0311
PED (mm Hg)^a^	9.9 ± 0.3	8.7 ± 0.4*	.0266
LVDP (mm Hg)^a^	57.2 ± 2.1	40.4 ± 4.1*	.0032
RPP (mm Hg × min^−1^ × 10^3^)^a^	17.4 ± 1.0	10.0 ± 1.0*	<.0001
Recovery (%)^c^	105.5 ± 10.2	74.5 ± 5.3*	.0040

Abbreviations: CF, coronary flow; dP/dt_max_, baseline left ventricular maximal contractility; dP/dt_min_, baseline left ventricular maximal relaxation; HR, heart rate; I/R, ischaemia/reperfusion; LVDP, left ventricular developed pressure, PED, left ventricular end‐diastolic pressure; n = 4‐14 *P < .05 vs Aerobic control; RPP, rate pressure product.

^a^ After I/R (77 min of the experiment).

^b^ After ischaemia (first minute of reperfusion).

^c^ The difference between RPP in 25 and 77 min of the experiment.

### 3.3 Klotho protein in the rat hearts during I/R injury

**FIGURE 5:**
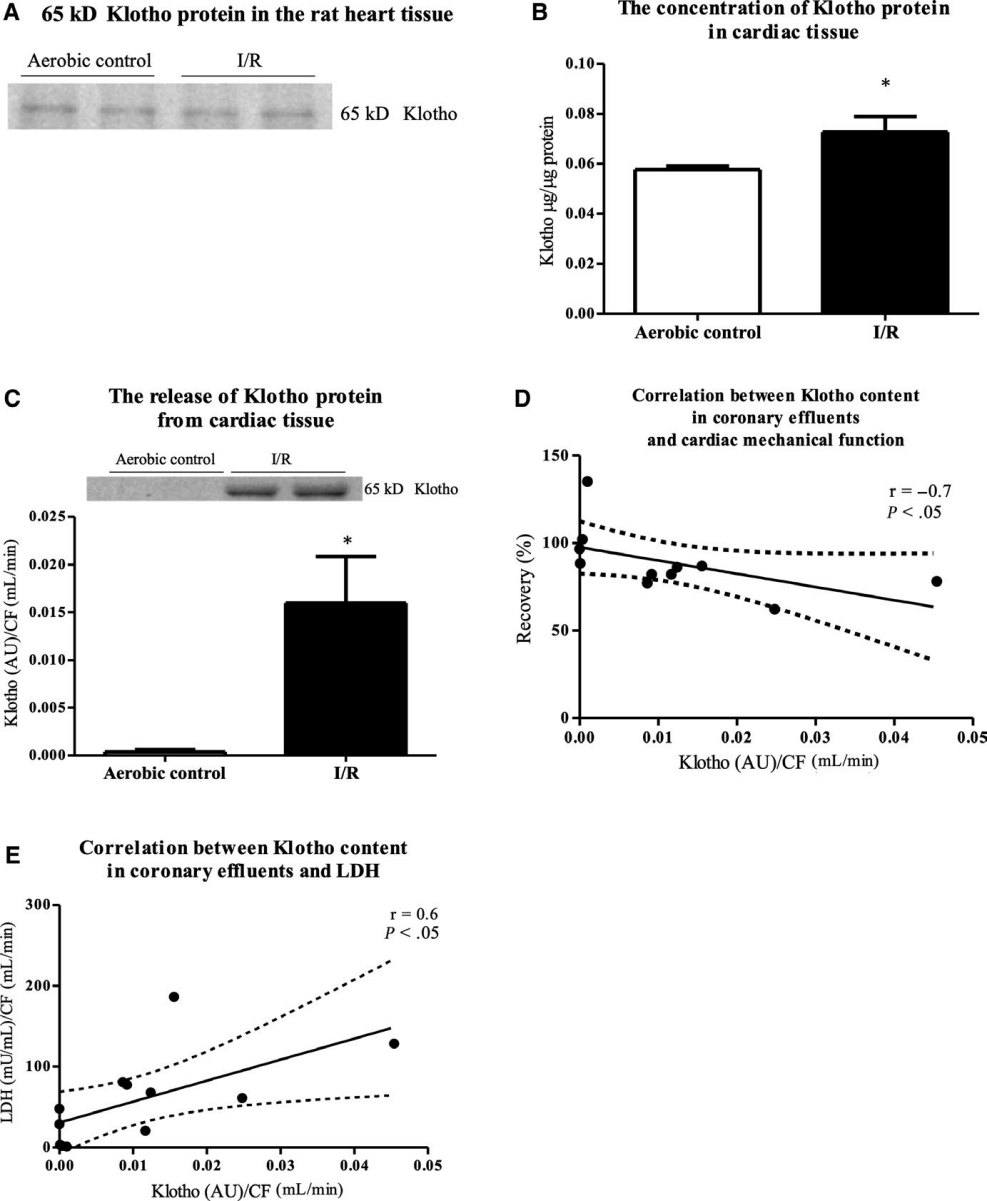
The expression and release of Klotho protein from the rat heart tissue under I/R injury. A, 65 kDa bands of Klotho protein in the rat heart tissue. Qualitative examination by Western blot. B, The concentration of Klotho protein in the rat heart tissue by ELISA. Klotho protein concentration in tissue homogenates was normalized to total protein concentration. C, Klotho content in coronary effluents (collected at the beginning of reperfusion) by Western blot. The content of Klotho protein in coronary effluents was expressed as AU and normalized to CF. D, Correlation between Klotho content in coronary effluents and cardiac mechanical function and E, LDH level. AU, arbitrary units; CF, coronary flow; LDH, lactate dehydrogenase; mU/mL, milli international enzyme units per millilitre; *P < .05 vs aerobic control; mean ± SEM; n = 4‐8

To document the expression of Klotho protein at the tissue level, an isolated rat heart model was used. Immunoblot analysis showed that Klotho protein was expressed in the rat heart tissue as a band with apparent molecular mass at approximately 65 kDa (Figure 5A). There was a significantly increased tissue expression of Klotho protein and its release to coronary effluents in I/R group in comparison with aerobic group (Figure 5B,C). The content of Klotho in coronary effluents negatively correlated with cardiac mechanical function (*P* < .05, *r* = −.7) and positively correlated with LDH level (*P* < .05, *r* = .6) (Figure 5D,E).

### 3.4 An influence of Klotho protein on cell viability

**FIGURE 6:**
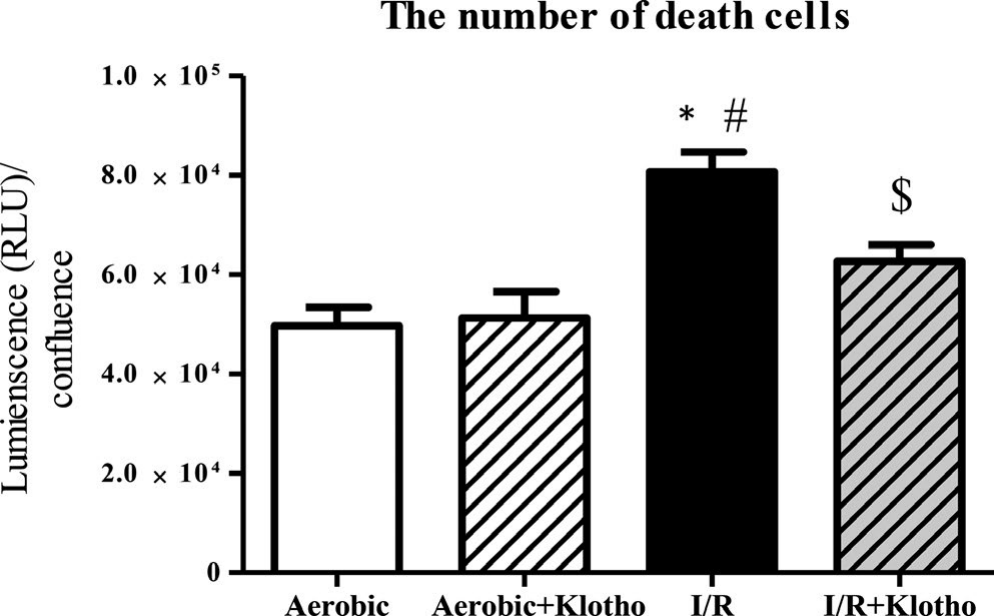
An influence of Klotho protein on cell viability. The number of death cells in the presence and absence of recombinant Klotho protein (1 μg/mL) by luminescent cytotoxicity assay. The data were normalized to cell confluence and expressed in RLU. The viability of myocytes was based on the number of death cells. I/R, ischaemia/reperfusion; RLU, relative light units; *P < .05 vs aerobic control; ^#^ *P* < .05 vs aerobic control + Klotho; ^$^ *P* < .05 vs I/R; mean ± SEM; n = 8

To check the potential cardioprotective effect of Klotho protein, HCM were subjected to I/R in the presence and absence of Klotho protein. The treatment of cardiomyocytes with recombinant human Klotho (1 µg/mL) resulted in increased viability of cells during I/R. To check the toxicity of Klotho protein on cardiomyocytes, cells from aerobic control group were incubated with Klotho. Supplementation of recombinant Klotho protein decreased the number of dead cells in I/R group in comparison with I/R group without Klotho addition and did not show toxicity in aerobic control group (Figure 6).

### 3.5 An influence of Klotho protein on metabolic activity of cardiomyocytes

**FIGURE 7:**
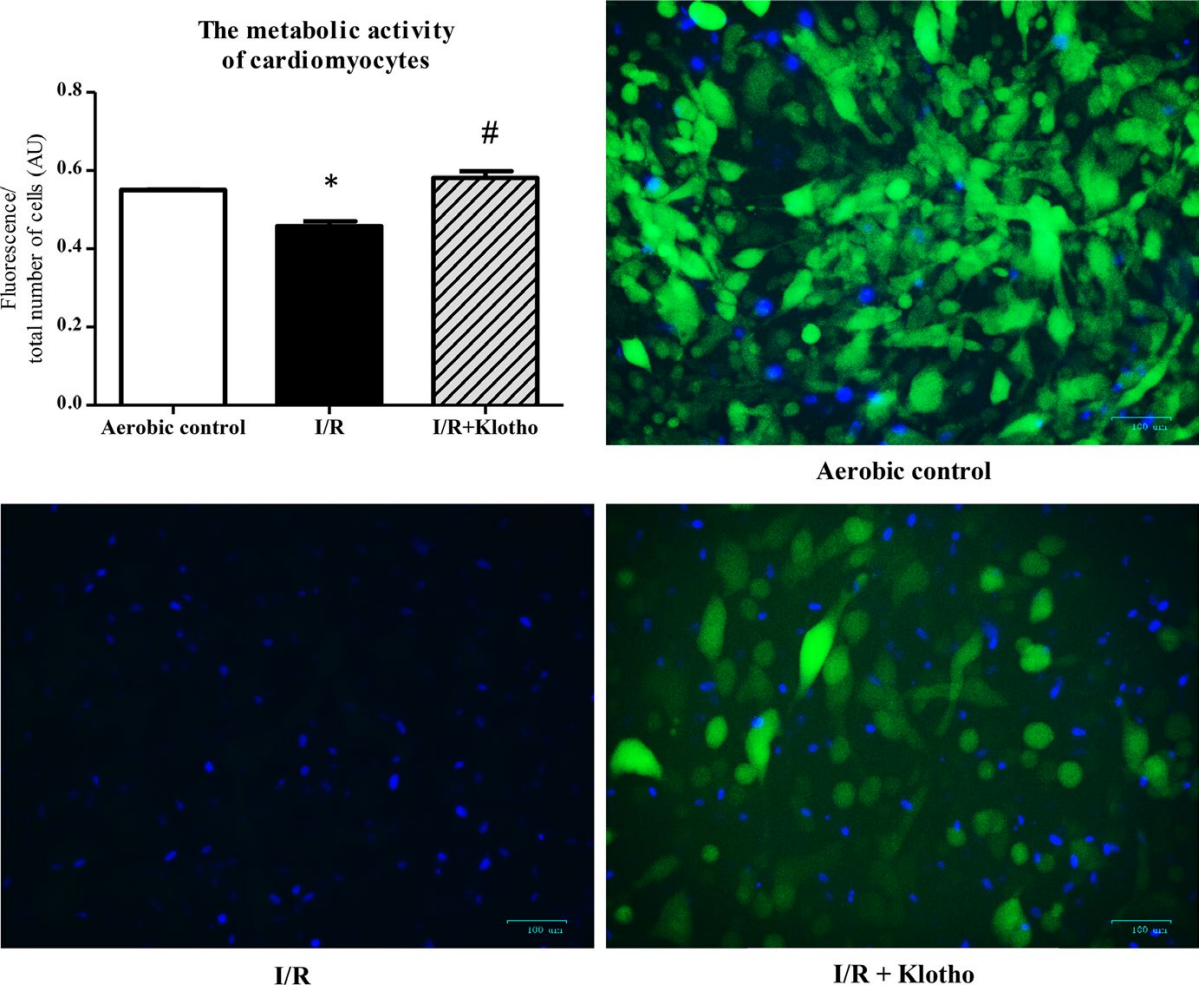
An influence of Klotho protein on metabolic activity of cardiomyocytes subjected to I/R. Fluorescence microscopy: cells that stained with FDA (green fluorescence) and excluded DAPI (blue fluorescence) were considered viable, whereas cells that excluded FDA and stained with DAPI were considered necrotic. To determine the metabolic activity of cells, green fluorescence (live cells) was divided by the total number of cells (green + blue fluorescence). Graph bars show the average of total fluorescence of cells in each experiment. Magnification 200×; scale bar = 100 μm; AU, arbitrary unit; DAPI, 4',6‐diamidino‐2‐phenylindole; FDA, fluorescein diacetate; I/R, ischaemia/reperfusion; *P < .05 vs aerobic control, ^#^ *P* < .05 vs I/R; mean ± SEM; n = 3‐7

To explore an impact of Klotho on the metabolic activity of cardiomyocytes, the vital inclusion dye FDA and the vital exclusion dye DAPI were used. Cells that stained with FDA and excluded DAPI were considered viable, whereas cells that excluded FDA and stained with DAPI were considered necrotic. I/R injury led to decreased metabolic activity of the cardiomyocytes. The treatment with Klotho protein (1 µg/mL) significantly increased metabolic activity of the cardiomyocytes that underwent I/R in comparison with Klotho untreated I/R group (Figure 7).

## 4 DISCUSSION

As the discovery of Klotho, a significant amount of efforts has been devoted to characterize its protective role in oxidative stress, inflammation, fibrosis and apoptosis.^6^, ^9^, ^10^, ^11^, ^23^ It is well established that Klotho acts prophylactic and therapeutic in acute renal failure or chronic kidney disease.^12^, ^14^, ^24^ Therefore, the main aim of this study was to identify the role of Klotho protein in the ischaemic injury of heart. In the present study, the compensatory production and release of Klotho protein from cardiomyocytes after ischaemic damage were shown. Our research confirmed that Klotho protein effectively reduced damage and apoptosis of the cardiomyocytes during I/R, showing cardioprotection. We strongly suggest that Klotho protein can be valuable both as a marker of injury and as a potential cardioprotective agent.

There are 3 types of Klotho protein: membrane‐bound (135 kDa), shed and secreted. It is known that membrane proteases cut membrane‐bound Klotho to generate shed types of Klotho: full‐length 130 kDa molecule (the major product of shedding) and a fragment of molecular mass of about 68 kDa. The alternative transcriptional termination of Klotho gene leads to production of secreted Klotho (about 65 kDa). Soluble types of Klotho protein (shed and secreted) are found in the blood, urine and cerebrospinal fluid.^25^ Our research showed that Klotho protein is highly expressed in human cardiomyocytes and at the tissue level in rat hearts. We detected Klotho protein in the heart and coronary effluents at apparent molecular mass of about 65‐70 kDa. Similarly, other investigators showed that Klotho protein was expressed at molecular mass of about 65 kDa in adipose‐derived mice stem cells (ADSCs) and in human pancreatic β‐cells, while full‐length Klotho protein (130 kDa) was not detectable.^26^, ^27^ We speculate it is due to activity of the membrane proteases ADAM10 and ADAM17 (a disintegrin and metalloproteinase domain‐containing proteins 10 and 17) that cut membrane‐bound Klotho to create the proteins with molecular mass of about 65 kDa.^28^ Moreover, it was shown that the expression of secreted 65 kDa Klotho predominates over the production of shed 68 kDa protein.^29^ It could explain that the major form of Klotho detected in our research was protein at apparent molecular mass of about 65‐70 kDa. It should be also noted that there are some difficulties in the measurement of Klotho types and making a distinction between shed and secreted forms due to the highly conserved sequences and similarity in molecular mass. It is possible to measure only total soluble Klotho using currently available commercial tests for the quantitative analysis like ELISA. We expect technical advances will allow these key details to be filled in the future.

A large number of studies over last decade have greatly enriched our knowledge of correlation between Klotho deficiency and risk of CVD.^30^, ^31^, ^32^, ^33^ Here, we showed that expression of Klotho mRNA was elevated in the cardiomyocytes subjected to I/R injury. An increased expression of Klotho mRNA was accompanied by enhanced Klotho protein synthesis and cell surface expression. Moreover, an enhanced production of Klotho protein was observed also at the tissue level in hearts that underwent global no‐flow ischaemia. Our results suggest Klotho involvement in the process of oxidative stress and apoptosis during I/R. Given that, we propose a compensative production of Klotho in the cardiomyocytes under stress‐related conditions to protect cardiac tissue against ischaemic damage. Interestingly, Klotho level was depleted in the pancreatic islets in patients with type 2 diabetes mellitus.^27^ The compensatory production of Klotho, thus renoprotection, has not been found also in renal disorders. It was reported that renal I/R injury reduced Klotho levels in the kidney tissue and serum in animal models.^34^, ^35^, ^36^ Similarly, the renal transplant recipients who developed delayed graft function (DGF) showed a dramatic reduction in renal and plasma Klotho levels.^35^ It was shown that Klotho deficiency acts as an early biomarker for kidney damage and contributes to progression of chronic kidney disease. Thus, there is no renoprotection due to reduced expression of renal Klotho in the state of kidney disorders.^36^, ^37^, ^38^, ^39^ Surprisingly, scientists have found depletion of renal Klotho level in the state of hypertension, diabetes and renal failure, but not after acute myocardial infarction in rats.^40^ Furthermore, there was an association between higher serum Klotho level and reduced occurrence of cardiovascular events and cardiovascular death in human.^41^ The patients after MI had elevated level of serum Klotho, which suggests compensatory production of Klotho to prevent the development of subsequent heart lesions.^42^ As MI is recognized as an important cause of cardiac hypertrophy and remodelling development, the compensative expression of Klotho after cardiac injury could protect against left ventricular hypertrophy (LVH) or additional heart failures.^43^ Considering our results, we speculate that there is a compensative production of cardiac Klotho during heart disorders to reduce the area of injury and to protect cardiac tissue from damage and further lesions, like heart hypertrophy and remodelling. We also suggest that adequate expression of Klotho is essential for recovery of proper heart function. Preserved Klotho expression may support cardiovascular protection and serve as a prognostic tool and therapeutic target for cardiovascular diseases.

In our study, we showed that Klotho protein was intensively released from cardiac tissue subjected to ischaemic damage. Thus, we propose that enhanced expression of Klotho, its further release from cardiomyocytes into extracellular space and increased level in the body fluids may be useful as a marker of ischaemic heart injury. This hypothesis can be supported by the evidence of increased serum Klotho level in patients after heart failure.^42^ Interestingly, there are investigations demonstrating Klotho deficiency as a biomarker for kidney injury and for number of cancers, including breast cancer, lung cancer or hepatocellular carcinoma. They showed decrease in both plasma and urine Klotho protein levels in animal kidney injury models.^34^, ^39^, ^44^ However, in our investigation, ischaemic heart injury and reduced mechanical function of the heart were related to the high content of Klotho in coronary effluents. It may be due to the activity of membrane proteases that are responsible for cutting of membrane‐bound Klotho and/or enhanced compensative expression of Klotho during ischaemic damage. As a result of alternative splicing of Klotho gene or shedding of membrane‐bound Klotho, the various kinds of Klotho protein forms are released to extracellular space. Taking together, we suggest that compensative production of Klotho and its release from injured cardiac cells may be valuable as a marker of heart damage.

Research from the last few years showed protective effect of Klotho overexpression or administration during ischaemic injury of the brain and kidneys.^13^, ^34^ Thus, we studied if Klotho administration may mitigate I/R injury also in the cardiac cells. Our results revealed that Klotho supported the metabolic function of cardiomyocytes and decreased ischaemic damage, thus had cardioprotective action. It may be due to protection from death caused by oxidative stress and induction of oxidative stress resistance by Klotho. Importantly, several studies have previously proved the antioxidative, antiapoptotic and antifibrotic activity of Klotho.^9^, ^17^, ^43^, ^45^ These findings confirm the beneficial influence of Klotho on cell metabolism and survival observed in our research. Researchers emphasize that recombinant Klotho may be prophylactic and therapeutic in progression of acute to chronic kidney disease, renal fibrosis and uremic cardiomyopathy.^14^, ^37^ Moreover, it was reported that Klotho reduced apoptosis in experimental ischaemic acute kidney injury or renal and cerebral I/R injury.^13^, ^24^, ^36^ Therefore, we propose that Klotho may be considered as a potential therapeutic agent also in I/R injury of heart in the future.

## 5 SUMMARY

Our research showed that: (a) there is an increased compensative production of Klotho protein in the cardiomyocytes during I/R to protect ischaemic heart from further injury; (b) an enhanced production of Klotho during I/R and its release to extracellular space can be used as a marker of heart damage; and (c) Klotho administration protects the cardiomyocytes against I/R injury and improves their metabolism after ischaemic damage.

In conclusion, this study confirmed the potential cardioprotective role of Klotho in the development of I/R injury. We propose that administration of Klotho protein into hearts may prevent the cardiomyocytes from the damage or reduce the area of injury. We strongly suggest that Klotho protein can be valuable both as a marker of injury and as a potential cardioprotective agent and contributes to compensatory mechanism which mitigates the initial damage under cardiac stress.

We are also aware that further studies on the role of exogenous Klotho on cardioprotection on in vivo model are needed. So the next goal of our study is to check whether administration of Klotho before I/R or during I/R will be cardioprotective, and how this cardioprotection is induced.

## CONFLICT OF INTEREST

The authors confirm that there are no conflicts of interest.

## AUTHORS' CONTRIBUTIONS

All authors participated in the design, interpretation of the studies, analysis of the data and review of the manuscript; AO, AKZ and IBL designed the research study; AO, AKZ, MB and IBL performed the research; AO and IBL analysed the data; AO wrote the paper; IBL made critical revision of the manuscript.

## Data Availability

The data that support the findings of this study are available from the corresponding author upon reasonable request.
